# Experimental Investigation on Macroscopic and Microscopic Mechanical Properties of Geopolymer-Stabilized Macadam

**DOI:** 10.3390/ma18020454

**Published:** 2025-01-20

**Authors:** Hancheng Dan, Shenglong Ma, Mengjin Li, Jiawei Tan, Haoran Zhang

**Affiliations:** 1 School of Civil Engineering, Central South University, Changsha 410075, China; 2 National Engineering Research Center of High-Speed Railway Construction Technology, Central South University, Changsha 410075, China; 3 School of Civil and Environmental Engineering, Nanyang Technological University, Singapore 639798, Singapore

**Keywords:** cement-stabilized macadam, geopolymer-stabilized macadam, pavement, permanent deformation, interface

## Abstract

Geopolymer, as a promising inorganic binding material, holds potential for use in constructing base layers for highway pavements. This study aims to evaluate the mechanical properties of geopolymer-stabilized macadam (GSM) at both the micro- and macro-scale by a series of tests, demonstrating that high-Ca GSM is a high-quality material for pavement base layers. The results demonstrated that GSM exhibits outstanding mechanical and fatigue properties, significantly surpassing those of cement-stabilized macadam (CSM). Performance improvements were particularly notable with higher binder-to-aggregate ratios. GSM derived from a high-Ca precursor achieved a relatively higher fatigue life and resistance to permanent deformation under cyclic loading, outperforming CSM. Furthermore, relationship models developed from the indirect tensile fatigue test results provide a valuable framework for evaluating GSM’s long-term road performance. Microstructural analyses revealed that geopolymer features a reticulated gel structure and a denser, more continuous internal matrix, which contribute to its superior properties. The interface products of GSM, including C–A–S–H gel and C(N)–A–S–H gel, enhance mechanical interlocking and promote early strength development, accounting for its exceptional mechanical strength and fatigue resistance. These findings offer valuable insights and technical guidance for employing geopolymer as a sustainable and effective alternative to cement-stabilized macadam in base layer construction.

## 1. Introduction

The road pavement base endures repeated stress from traffic loads. Over time, the accumulated irreversible deformation from repeated tensile stress can lead to material failure through fatigue cracking [1]. Numerous studies underscore the critical role of fatigue performance in base-stabilizing materials, highlighting the need for materials that can withstand repeated critical loads and minimize permanent deformation throughout their service life [2,3,4,5]. Currently, cement-stabilized crushed stone (CSM) is widely used in pavement base construction. However, due to its heterogeneous nature and relatively low cement content, CSM often suffers from inadequate hydration, leaving many pores unfilled. This lack of complete hydration compromises the material’s volume stability and fatigue resistance [6]. Consequently, cracking is prevalent in CSM, signaling a need for enhanced crack resistance to improve road performance. Furthermore, the high energy consumption and substantial greenhouse gas emissions associated with cement have become increasingly concerning. In light of the urgent need to reduce carbon emissions, these issues have spurred researchers to explore alternative materials for road base construction.

Geopolymer is a type of cementitious material created by reacting aluminosilicate sources (such as fly ash, metakaolin, and slag) with alkali or acid activators. Renowned for their high strength, durability, and low carbon footprint [7,8,9,10,11,12], geopolymers have gained significant academic attention as promising alternatives to cement. Additionally, the compatibility of geopolymer materials with existing road materials—such as similar elastic modulus and Poisson’s ratio—has driven many studies investigating their application in road engineering. Numerous studies have highlighted the significance of utilizing metakaolin (MK)-ground granulated blast furnace slag (GGBS)-based geopolymer in road engineering to produce stabilized materials with a more compact microstructure, enhanced load-bearing capacity, and improved durability for pavement base layers [13,14,15]. For example, Feng et al. studied the microstructure of granulated blast furnace slag (GBFS)-based geopolymer incorporating metakaolin using methods such as Fourier Transform Infrared Spectrometer (FTIR) and X-Ray Diffraction (XRD). It was found that metakaolin can enhance the interactions between gel products and induce more chemical reactions, thereby increasing strength [16]. Shi et al. found that using fly ash-based geopolymer to replace part of the cement as cementitious materials is an effective way to improve the resistance to fatigue cyclic loading of CSM [17]. This is because geopolymer-stabilized materials have a more compact microstructure. Some studies have also found this characteristic through Scanning Electron Microscope (SEM) testing [14,18,19,20].

Variations in the microstructural composition and mechanical properties of cement-based composites significantly influence their overall mechanical behavior [6]. This is particularly true for CSM, which is inherently heterogeneous in structure. The microstructural properties of CSM play a crucial role in determining its mechanical performance and cannot be overlooked [21,22,23]. However, limited research has examined the changes in mechanical behavior at both the micro- and macro-scale when cement in CSM is replaced with geopolymers. Doan et al. [24] investigated the strength of fly ash–slag-based geopolymer-stabilized soil under varying precursor material dosages and concluded that these materials are suitable for use in pavement base layers. Anburuvel et al. [25] explored the feasibility of geopolymer-stabilized soil as a road material from the perspectives of compressive strength and environmental impact. Similarly, Arulrajah et al. [13] conducted mechanical strength tests and microstructural analyses on fly ash- and slag-based geopolymer-stabilized materials but did not address their fatigue performance. The microscopic structure and reaction products at the interface between the binder and aggregate can be observed using SEM, EDS, and FTIR. Furthermore, current research predominantly focuses on the compressive strength and microstructure of geopolymer-stabilized materials. However, the vertical compressive stress and horizontal tensile stress induced by traffic loads in pavement subbase layers [26], combined with the microstructural heterogeneity of geopolymer-stabilized materials, make it challenging to fully evaluate the fatigue failure characteristics of these materials under cyclic loading conditions. Repeated tensile stresses cause permanent deformation, ultimately resulting in fatigue cracking failure [1]. Hoy et al. [27] addressed this issue by assessing the elasticity and fatigue performance of cement-stabilized recycled materials under traffic loading, monitoring permanent deformation during indirect tensile fatigue tests. Therefore, evaluating the macroscopic mechanical properties of geopolymer-stabilized macadam (GSM) can be achieved by monitoring the deformation development characteristics of specimens during the indirect tensile fatigue testing process.

**Figure 1:**
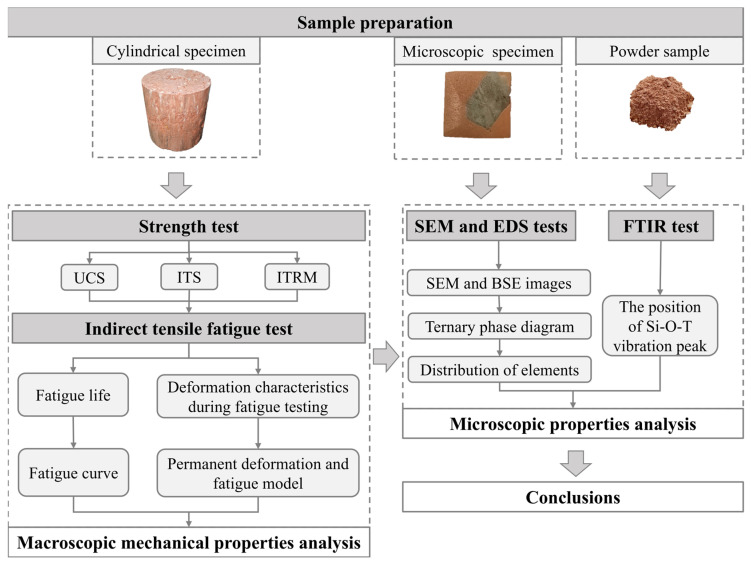
Technical approach.

To address the research gap, the present study aims to evaluate the mechanical properties of GSM at both the micro- and macro-scales by a series of tests. Linear Variable Differential Transformer (LVDT) displacement sensors will first monitor strain development in GSM during fatigue loading to assess damage macroscopically in the indirect tensile fatigue test. The overall mechanical properties of geopolymer composites will be evaluated by examining the relationships among unconfined compressive strength (UCS), splitting tensile strength (ITS), indirect tensile rebound modulus (ITRM), permanent deformation, and fatigue life. Subsequently, the microstructural and chemical characteristics of the interfacial transition zone (ITZ) in GSM will be examined using FTIR, SEM, and EDS to uncover the micro-scale mechanisms contributing to the macroscopic mechanical differences between CSM and GSM materials. The technical approach of this paper is illustrated in Figure 1.

## 2. Materials and Methods

### 2.1. Raw Materials

**Figure 2:**
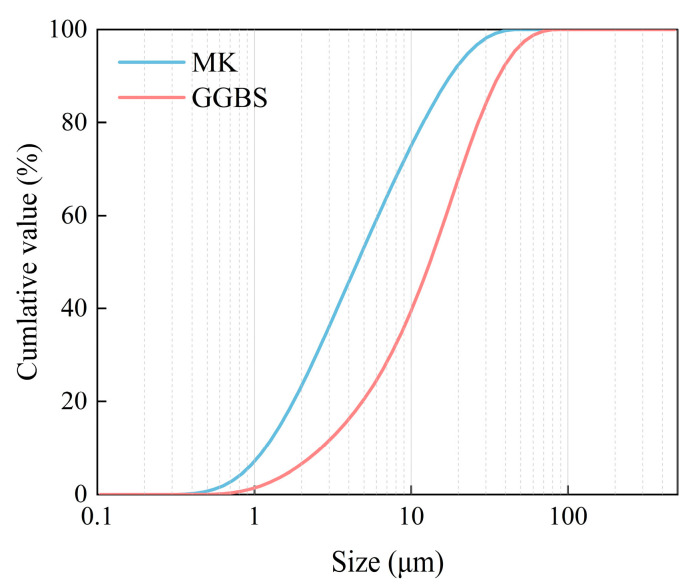
Particle size distribution of precursor materials.

Low-Ca precursor, i.e., metakaolin (MK), and high-Ca precursor, i.e., ground granulated blast furnace slag (GGBS), were chosen as the raw materials for geopolymer synthesis. The average particle size (D50) of MK and GGBS was 4.5 μm and 13.17 μm, respectively. Table 1 and Figure 2 show the chemical composition and particle size distribution of these materials, respectively. Limestone aggregate was used to prepare the stabilizing material. The physical properties of the limestone aggregate can be found in Table 2.

The activator was prepared by dissolving NaOH in water and stirring thoroughly to form a NaOH solution (11.68 mol/L). The modulus of the water glass is 3.3. This NaOH solution was then cooled for 24 h before adding Na_2_SiO_3_. The following proportions were selected for geopolymer synthesis: a Na_2_O-to-binder ratio of 8%, an SiO_2_-to-Na_2_O molar ratio of 1.5, and a water-to-binder ratio of 0.4. These proportions were chosen based on preliminary test results.

### 2.2. Sample Preparation

Based on the pre-test results, the test groups were organized as presented in Table 3. G3, G4, and G5, which use GGBS as the main precursor material, are referred to as high-Ca GSM. M4, which uses MK as the main precursor material, is classified as low-Ca GSM, while P4 is designated as CSM.

**Figure 3:**
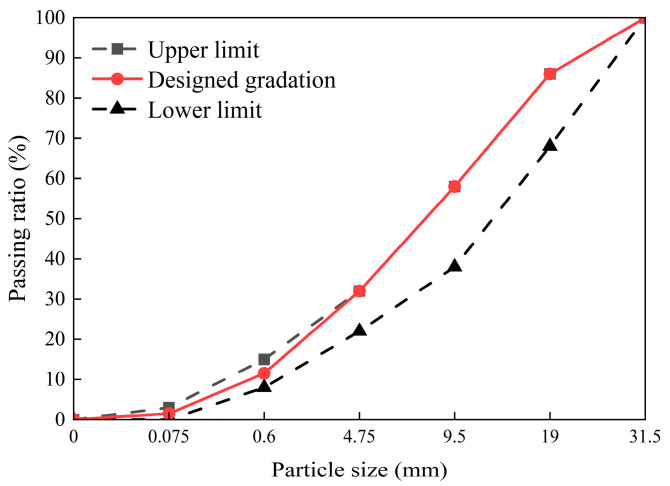
Designed gradation curve.

Samples were prepared following the procedure outlined below: First, GGBS and MK powders were mixed at low speed for 2 min to ensure a uniform precursor material. Next, the alkali activator was added. To prevent splashing of liquids and powders, the precursor material and alkali activator were initially mixed at low speed for 2 min, followed by high-speed mixing for an additional 2 min to thoroughly combine the geopolymer paste. Aggregate gradation was based on the C–B–3 gradation recommended in the Technical Guidelines for Construction of Highway Roadbases (JTG/T F20-2015) [29], with the designed gradation curve shown in Figure 3. The maximum dry density and optimum moisture content were determined using the heavy-duty compaction test as specified in JTG E51-2009 [30], with results presented in Table 3. Cylindrical specimens (150 mm in diameter and 150 mm in height) were formed using hydrostatic compression. The specimens were then sealed in plastic bags and stored in a curing box maintained at 20 °C ± 2 °C with humidity levels above 95% until the designated test age. On the final day of the curing period, the specimens were immersed in water.

### 2.3. Strength Test

According to JTG E51-2009 [30], the UCS of the cylindrical specimens was tested using the press machine with a loading rate of 1 mm/min. The maximum pressure at the time of specimen failure was recorded, and the UCS was calculated using Equation (1). The ITS of the cylindrical specimen was tested using the UTM testing machine at a loading rate of 1 mm/min. The splitting fixture used in the test met the requirements of JTG E51-2009. The maximum pressure at the time of specimen failure was recorded, and the ITS was calculated using Equation (2). The ITRM was tested in accordance with JTG E51-2009 using the pavement material strength tester, manufactured by Lushida (Shanghai) Instrument Equipment Co., Ltd. in Shanghai, China. Six levels of load were continuously applied, each within 70% of the destructive load. The deformation value of the cylindrical specimen was recorded when the load was applied for 1 min. The recovery value of the cylindrical specimen’s deformation was recorded 0.5 min after unloading, and the next load was then applied. The ITRM was calculated using Equation (3).

(1) $$R_{c}=\frac{4P}{\pi d^{2}}$$

where $R_{C}$ is the unconfined compressive strength of the specimen (MPa); d is the diameter of the specimen (mm); and P is the maximum pressure when the specimen is destroyed (N).

(2) $$R_{i}=\frac{2P}{\pi dh}(\sin2\alpha -\frac{a}{b})$$

where $R_{i}$ is the ITS of the specimen (MPa); a is the width of the indentation strip (mm); α is the angle of the center of the circle corresponding to the width of the half−indentation strip (°); d is the diameter of the specimen (mm); P is the maximum pressure at the time of destruction of the specimen (N); and h is the height of the specimen after immersion in water (mm).

(3) $$E_{i}=\frac{p-p_{0}}{dl_{x}}(0.27+1.0\mu )$$

where $E_{i}$ is the indirect tensile resilience modulus (MPa); p is the load at each level (N); $p_{0}$ is the initial load at each level (N); d is the diameter of the specimen (mm); $l_{x}$ is the horizontal resilience deformation (mm); and μ is the Poisson’s ratio, 0.25.

### 2.4. Indirect Tensile Fatigue Test

The stress paths observed in the indirect tensile fatigue test of cylindrical specimens—vertical compression and horizontal tension—are analogous to the stress patterns experienced by the pavement base layer [22]. The indirect tensile fatigue test is recommended as an alternative to the flexural beam fatigue test due to the challenges associated with preparing beam specimens of cement-stabilized materials that have a low binder-to-aggregate ratio. The reproducibility and reliability of determining fatigue life and strength damage parameters for cement-stabilized materials and asphalt mixtures through indirect tensile fatigue testing have been well demonstrated [31,32,33,34].

**Figure 4:**
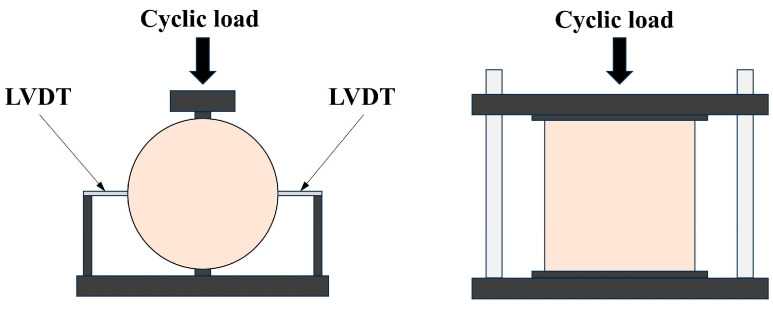
Schematic diagram of the indirect tensile fatigue test setup.

Under normal traffic conditions, the frequency of carloads acting on the road surface is approximately 10 Hz. Therefore, a loading frequency of 10 Hz was employed for all the fatigue tests in this study, with the loading waveforms set to half-sinusoidal waves [30]. Indirect tensile fatigue tests were conducted using stress control at stress levels of 0.44, 0.52, 0.60, and 0.68. The loading apparatus used for these tests was a Universal Testing Machine (UTM), manufactured by IPC global Pty Ltd in Sydney, Australia. The fatigue tests adhered to the indirect tensile test method recommended in BS-EN-12697-24 [35] for cylindrical specimens. A schematic diagram of the indirect tensile fatigue test setup is shown in Figure 4.

The fatigue life is defined as the total number of load cycles required to failure of the specimen. The tensile strain at the center of the sample is calculated using the following equation [35]:

(4) $$\varepsilon_{0}=\left(\frac{2∆H}{d}\right)\times \left[\frac{1+3\mu }{4+\pi \times \mu -\pi }\right]$$

where $\varepsilon_{0}$ is the tensile microstrain at the center of the specimen; ∆H is the horizontal deformation (mm); and d is the specimen diameter (mm).

(5) $$\varepsilon_{p}=2.13\frac{∆p}{d}$$

where $\varepsilon_{p}$ is the plastic tensile microstrain at the center of the specimen, and ∆p is the plastic horizontal deformation (mm).

### 2.5. Microstructural Characterization Instrumentation Methods

**Figure 5:**
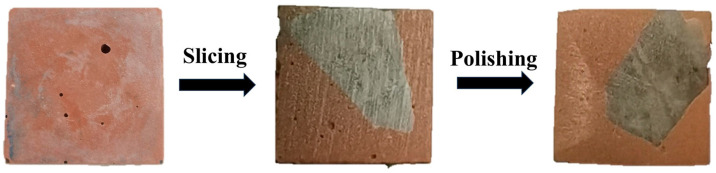
Microscopic specimen.

To analyze the microscopic morphological characteristics of the ITZ (interfacial transition zone) between the binder and aggregate, a detailed experimental study was conducted using a TESCAN MIRA LMS field emission SEM, manufactured by TESCAN ORSAY HOLDING, a.s. in Brno, Czech Republic. Samples for ITZ analysis were prepared as follows: The geopolymer paste was gradually injected into a 20 mm × 20 mm × 20 mm cube mold. The injection was paused halfway to add aggregates, after which the paste injection resumed until the mold was completely filled. After casting, the specimen was thoroughly vibrated and sealed with cling film. Following the procedure outlined in JTG E51-2009 [30], the specimen was cured in a standard curing box for 28 days. Once cured, the specimen was demolded and cut at the center using a Sodick WEDM-LS, manufactured by Sodick Co., Ltd. in Kanagawa, Japan, exposing a clear paste–aggregate bonding interface on the cut surface. The surface was then sanded and polished with silicon carbide sandpaper [36], as shown in Figure 5. EDS point scanning and line scanning of the interface samples were conducted with Xplore EDS (TESCAN ORSAY HOLDING, a.s., Brno, Czech Republic) to determine the elemental composition and concentration distribution within the ITZ. Prior to SEM and EDS testing, the observation surface of the interface sample was gold-coated to ensure high-quality imaging.

After 28 days of standard curing, the GSM and CSM paste was ground to a particle size of less than 200 mesh (74 μm) using an agate mortar and pestle. These powder samples were then subjected to FTIR analysis using a Thermo Scientific Nicolet 6700 instrument, manufactured by Thermo Fisher Scientific Inc. in Waltham, MA, USA.

## 3. Results and Discussions

### 3.1. Compressive Strength

**Figure 6:**
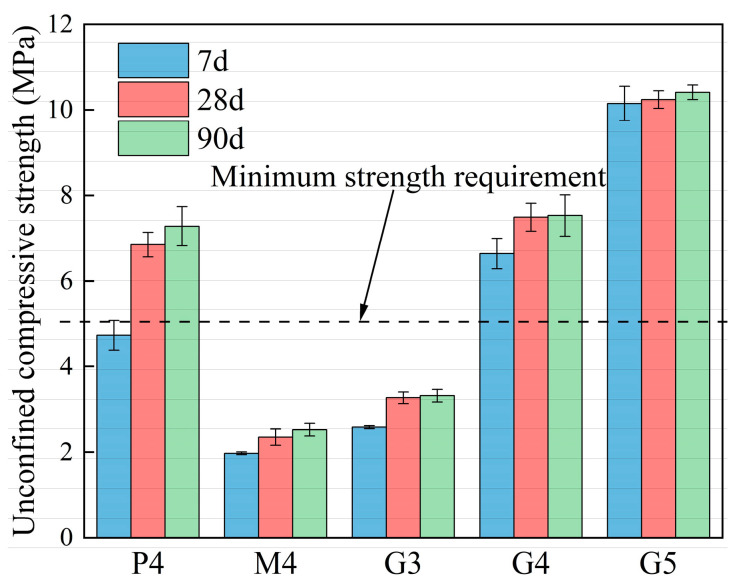
Test results of specimen UCS test (High-Ca GSM: G3, G4, and G5; low-Ca GSM: M4; CSM: P4).

Figure 6 presents the unconfined compressive strength (UCS) of CSM and GSM specimens at 7, 28, and 90 days. The results show that the UCS of both CSM and GSM increases over time, with a sharp rise observed between 7 and 28 days. Notably, the UCS of high-Ca GSM consistently surpassed that of CSM across all ages, while the UCS of low-Ca GSM remained lower than that of CSM at the same binder-to-aggregate ratio. According to JTG/T F20-2015, for very heavy, extra heavy, and heavy traffic loads, the 7-day UCS of cement-stabilized materials used for highway base layer repair should meet a minimum of 5 MPa and 4 MPa, respectively. These findings indicate that the high-Ca geopolymer achieves the desired high strength, with high-Ca GSM meeting the strength criteria required for highway base layer materials. Additionally, the UCS of high-Ca GSM increases rapidly in the early stages, with strength development essentially complete within 28 days. In contrast, the UCS of low-Ca GSM and CSM continues to grow more noticeably from 28 to 90 days, suggesting that a longer period is needed for full strength development.

**Figure 7:**
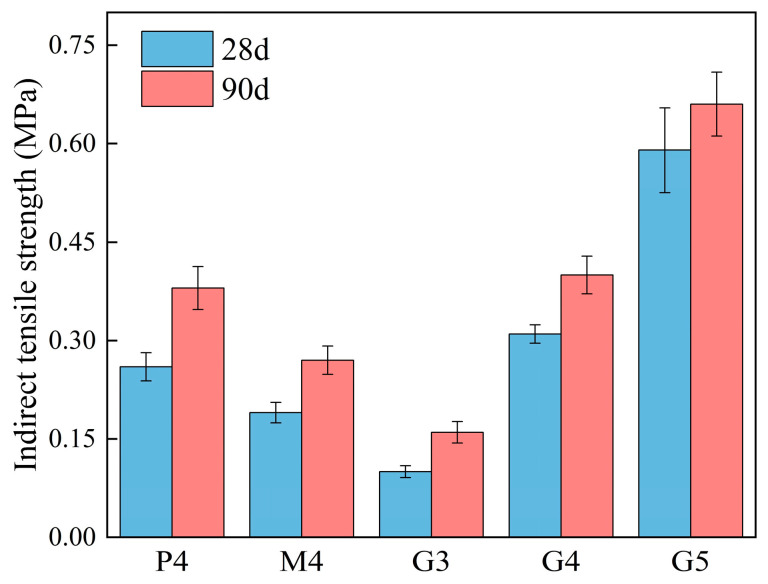
Indirect tensile strength test results of specimens (High-Ca GSM: G3, G4, and G5; low-Ca GSM: M4; CSM: P4).

In actual pavement applications, tensile stress issues often emerge first in the base layer. Therefore, the ability of base layer materials to withstand tensile stress is critical. Figure 7 shows the ITS test results for CSM and GSM specimens at 28 and 90 days. The ITS of all test groups increased with age. Notably, the ITS of high-Ca GSM exceeded that of CSM, while the ITS of low-Ca GSM was lower than that of CSM, given the same binder content. These ITS test results followed similar trends to those observed in the UCS tests. It is also worth noting that the ITS of the M4 test group surpassed that of the G3 test group, indicating a particular advantage of low-Ca GSM in resisting tensile stresses.

**Figure 8:**
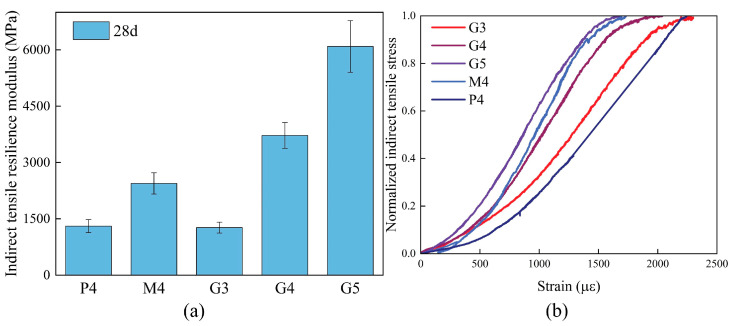
(**a**) Specimen indirect tensile resilience modulus test results; (**b**) Stress–strain relationship for indirect tensile tests with normalized treatment.

Figure 8a illustrates the ITRM of CSM and GSM at 28 days. Notably, with a constant binder-to-aggregate ratio, the resilience modulus of high-Ca GSM surpasses that of low-Ca GSM, while CSM exhibits the lowest resilience modulus, contrary to the trend observed in strength. The resilience modulus of cement-stabilized materials is influenced by their mechanical and physical properties [37,38], making it a crucial parameter in pavement structural design. Pavement service life is significantly impacted by fatigue cracking and rutting within structural layers. A higher resilience modulus in GSM indicates improved resistance to fatigue cracking and rutting caused by cyclic loading. Figure 8b shows stress versus strain plots recorded during the indirect tensile test at 28 days, with the indirect tensile stresses normalized. The trend of macrostrain at specimen failure aligns closely with the resilience modulus. CSM exhibits the highest strain at failure for the same binder-to-aggregate ratio, indicating good ductility, whereas low-Ca GSM shows the lowest strain at failure. As the binder-to-aggregate ratio increases, the fracture strain of high-Ca GSM gradually decreases, indicating reduced ductility.

### 3.2. Indirect Tensile Fatigue

#### 3.2.1. Indirect Tensile Fatigue Life

The phenomenological method primarily uses stress or strain as a parameter to develop fatigue prediction equations for semi-rigid materials. In this paper, the S–N fatigue equation shown in Equation (6) is derived using the phenomenological approach by fitting the material’s fatigue life under various stress levels [33].

(6) lgN=lgk−nlgt

where N is the fatigue life; t is the stress level; and k and n are coefficients.

**Figure 9:**
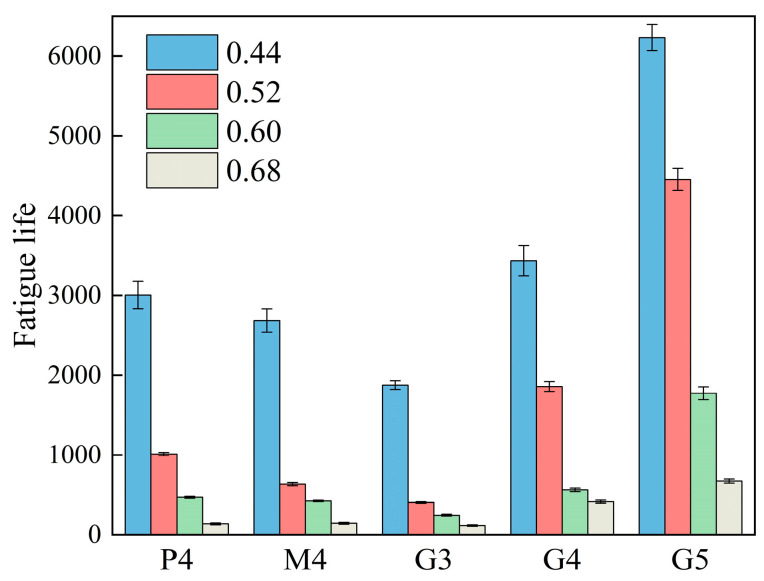
Indirect tensile fatigue life of different test groups of specimens at 0.44, 0.52, 0.60, and 0.68 stress levels.

**Figure 10:**
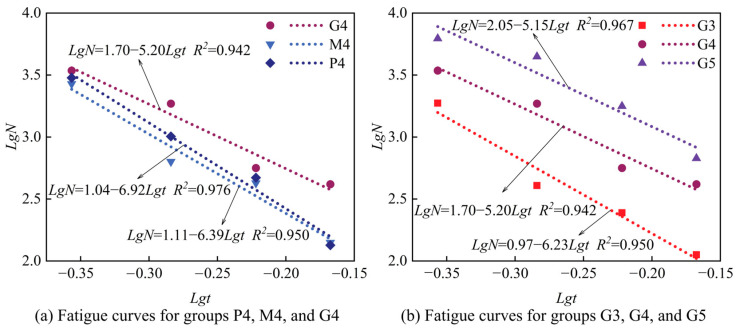
Indirect tensile fatigue curves for different test groups.

Based on the fatigue life test results (Figure 9), indirect tensile fatigue curves were derived by fitting the materials’ fatigue life under different stress levels, as shown in Figure 10. These fatigue curves display a strong linear correlation. Notably, in Figure 10a, the fatigue curves of the G4 group appear at the highest level for the same binder-to-aggregate ratio, indicating optimal fatigue performance of high-Ca GSM (G4). In comparison, low-Ca GSM (M4) shows slightly lower fatigue performance than CSM (P4). Within the high-Ca GSM test group (Figure 10b), the fatigue performance of G5 is superior to that of G4, while G3 shows the poorest fatigue performance. The positions of fatigue curves exhibit an upward trend with increasing binder-to-aggregate ratio, suggesting a gradual improvement in high-Ca GSM’s fatigue performance. This trend aligns with the strength test results (Figure 6), indicating consistent patterns between fatigue performance and strength. High-Ca GSM demonstrates the highest resilience modulus and fatigue life. As the binder-to-aggregate ratio increases, the density of high-Ca GSM also rises, leading to improved fatigue life and resilience modulus. This observation aligns with Festugato et al.’s findings on cement-treated sand, where resilience modulus and fatigue life increased with decreased porosity in cement-stabilized materials [39]. Indirect tensile fatigue test results suggest that high-Ca GSM outperforms CSM in fatigue performance, potentially extending pavement service life when used in road base layers. This advantage can be attributed to the higher density and strength of high-Ca GSM.

#### 3.2.2. Deformation Characteristics During Fatigue Testing

**Figure 11:**
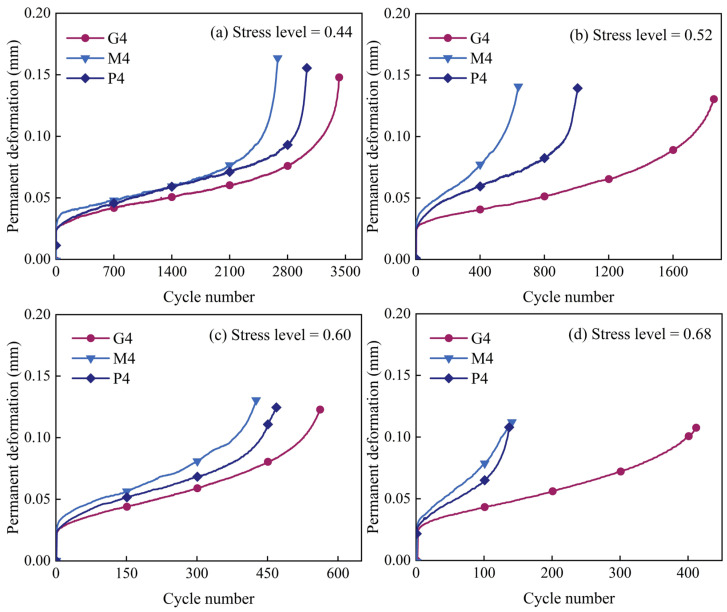
Permanent deformation versus number of load cycles for P4, M4, and G4.

Observation of stress and strain changes in the specimen during testing reveals that both increase with the applied load. However, upon load removal, stress returns to zero while strain does not fully recover. Under cyclic loading, the specimen undergoes both elastic deformation (recoverable upon load removal) and permanent deformation (irrecoverable upon load removal), with their sum representing total deformation. Permanent deformation in pavement structures can lead to issues such as cracking and rutting, reducing performance and service life. Therefore, this paper examines the relationship between the horizontal deformation of cement-stabilized and geopolymer-stabilized macadam and the number of load cycles. Figure 11 shows the relationship between permanent deformation and the number of load cycles at stress levels of 0.44, 0.52, 0.60, and 0.68 for the P4, M4, and G4 experimental groups. The graphs indicate that high-Ca GSM exhibits smaller permanent deformation than CSM, whereas low-Ca GSM shows larger permanent deformation than CSM at the same binder-to-aggregate ratio. This suggests that high-Ca GSM provides better resistance to structural deformation than traditional CSM, effectively reducing cracking and rutting caused by pavement structural deformation, thereby extending pavement service life and improving performance. This characteristic persists even with increasing load levels and can be attributed to the higher GGBS content in the precursor material, which leads to more C–A–S–H gel formation after alkali activation [40]. These gels offer greater strength and density compared with C–S–H gel (the primary hydration product of cement) and N–A–S–H gel (the primary product of low-calcium geopolymer), thereby enhancing fatigue resistance and the ability to withstand permanent deformation under cyclic loading. Similar results were reported by Shi et al., who found that incorporating GGBS-based geopolymers into cement-stabilized macadam generated more hydration products [17], which filled interfacial pores, increased aggregate–mortar adhesion, and subsequently improved fatigue properties.

**Figure 12:**
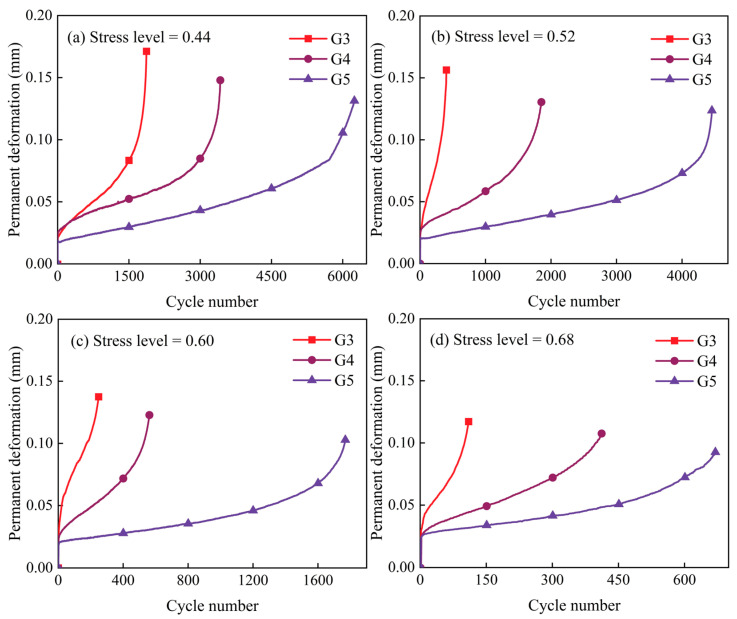
Permanent deformation versus number of load cycles for G3, G4, and G5.

Figure 12 shows the relationship between permanent deformation and the number of load cycles at stress levels of 0.44, 0.52, 0.60, and 0.68 for test groups G3, G4, and G5. It is evident that the permanent deformation of specimens decreases with an increase in geopolymer dosage in the high-Ca GSM test group with varying binder-to-aggregate ratios. This indicates a gradual enhancement in the specimens’ resistance to permanent deformation and an improvement in their fatigue resistance. This trend continues with higher loading levels. An increase in the binder-to-aggregate ratio directly correlates with a rise in the maximum dry density of the stabilized material, resulting in a denser internal structure and a notable reduction in microscopic cracks and defects. This densification contributes to improved fatigue resistance. Studies have shown that in cement-stabilized materials, increased density enhances resistance to deformation under cyclic loading and improves fatigue properties [27]. This finding confirms that replacing cement with high-Ca geopolymers as a binder for stabilizing macadam can extend the fatigue life of pavement base materials made with inorganic binders and reduce permanent deformation under cyclic traffic loading.

**Figure 13:**
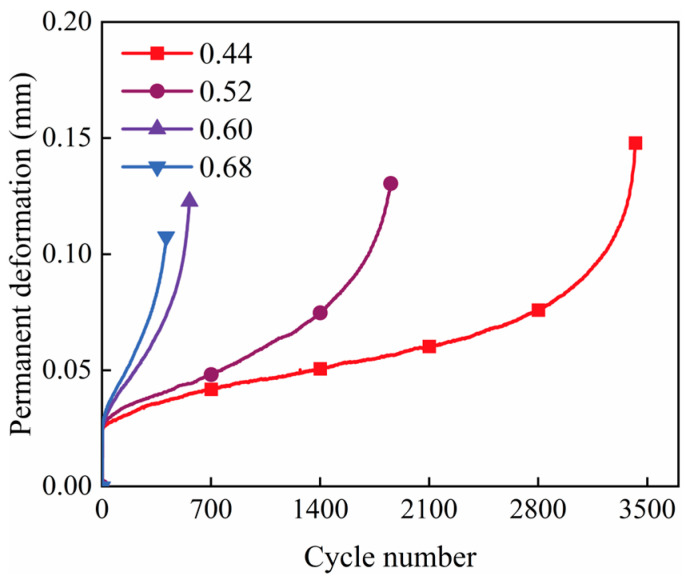
Curve of permanent deformation of G4 versus number of load cycles at different stress levels.

Additionally, observing the curves of permanent deformation for G4 against the number of load cycles at different stress levels (Figure 13) reveals that the permanent deformation of specimens increases more rapidly as the stress level rises. This phenomenon suggests a sudden failure condition in the material, where the brittleness of the stabilized material becomes increasingly evident, leading to a significant reduction in the road’s service life. Several studies on the fatigue properties of cement-stabilized recycled materials, cement-stabilized soils, and asphalt mixtures have shown a similar trend in plastic deformation at stress levels ranging from 0.3 to 0.75 [27,31,33,41,42,43,44]. Accumulated plastic deformation in road base structures under cyclic traffic loading is a primary cause of structural rutting in pavements, potentially leading to a loss of pavement serviceability [45]. Rutting and fatigue cracking are major concerns in road design, and reducing the permanent deformation of pavement structures under cyclic loading can help delay the onset of these issues. In designing pavement structures for heavy traffic conditions, using GSM with higher strength and greater resistance to permanent deformation as base materials can mitigate the risk of brittle failure in the base material under heavy traffic loads.

#### 3.2.3. Permanent Deformation and Fatigue Model

The gradual accumulation of permanent deformation in pavement structures under cyclic loading is a critical factor contributing to rutting and fatigue cracking. Numerous researchers have conducted fatigue tests on materials such as cement-stabilized soil, cement-stabilized aggregates, and recycled aggregates to evaluate the fatigue performance of inorganic bond-stabilized materials [17,27,31,46]. However, no studies have investigated the fatigue characteristics of MK-GGBS-based geopolymer-stabilized macadam, which is a key objective of this study.

**Figure 14:**
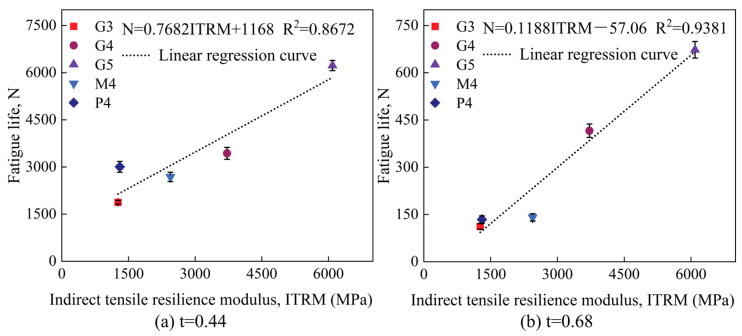
Relationship curves between fatigue life and indirect tensile resilience modulus for different test groups.

Based on the fatigue life and ITRM test results, we plotted the relationship curve between fatigue life and ITRM, as shown in Figure 14. The relationship curves for light traffic load (stress level t = 0.44) and heavy traffic load (stress level t = 0.68) are plotted separately, providing a foundation for predicting fatigue life based on resilience modulus.

**Figure 15:**
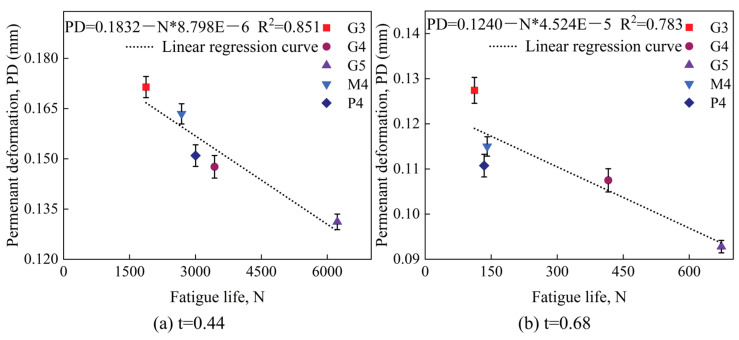
Relationship curves between maximum permanent deformation and fatigue life for different test groups.

Building on the fatigue test results, we developed the relationship curve between maximum permanent deformation and fatigue life, as shown in Figure 15. This curve is plotted separately for light traffic load (stress level t = 0.44) and heavy traffic load (stress level t = 0.68), providing a framework for predicting permanent deformation based on fatigue life.

**Figure 16:**
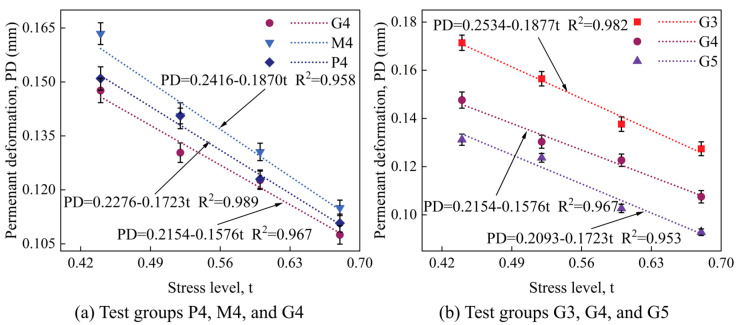
Relationship curves between maximum permanent deformation and stress level for different test groups.

Additionally, a prediction model for the maximum permanent deformation of geopolymer-stabilized macadam material under various loading levels was established, as depicted in Figure 16.

### 3.3. FTIR

**Figure 17:**
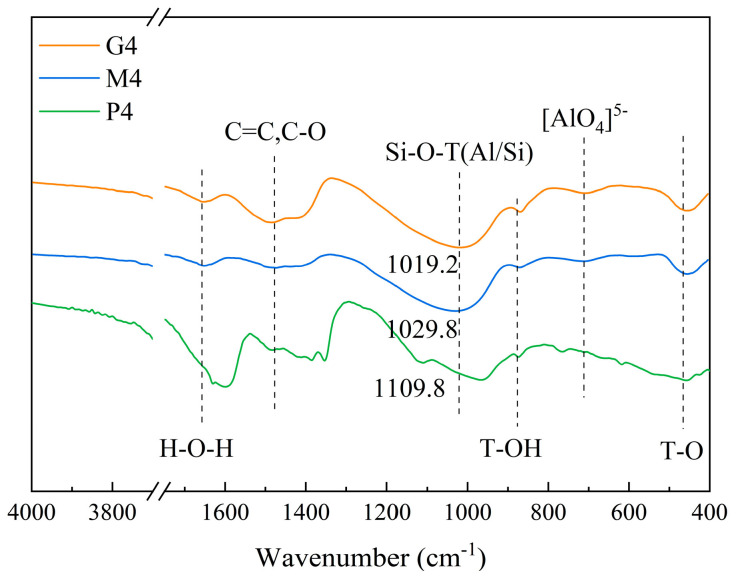
FTIR test results of the geopolymer and cement paste: G4 (high-Ca GSM), M4 (low-Ca GSM), and P4 (CSM).

Figure 17 shows the FTIR test results of the geopolymer and cement paste. The vibration peak at 1020 cm^−1^ is attributed to the asymmetric stretching vibration of Si–O–T (Al/Si) [47,48], indicating the formation of geopolymer gel. The vibration peak position of the geopolymer’s Si–O–T shifts towards lower wavenumbers compared with cement. The position of this Si–O–T vibration peak shifts to lower wavenumbers with increased GGBS content, moving from 1029.8 cm^−1^ for low-Ca geopolymer to 1019.2 cm^−1^ for high-Ca geopolymer. Since Si has a higher bond energy than Al in Si–O–T, the downward shift of the vibration peak suggests an increase in [AlO_4_]^5−^ substituting for [SiO_4_]^4−^ [49]. Greater involvement of [AlO_4_]^5−^ in the reaction results in the formation of a more uniform gel and promotes early strength development, consistent with the strength test results (Figure 6) in Section 3.1. This suggests that high-Ca geopolymer may have produced a larger amount of C–A–S–H gel [50,51], known for its denser structure compared with N–A–S–H and C–S–H gels [52]. The significant presence of C–A–S–H gel in the geopolymer products supports the superior strength and fatigue performance observed in high-Ca GSM.

### 3.4. SEM and EDS

**Figure 18:**
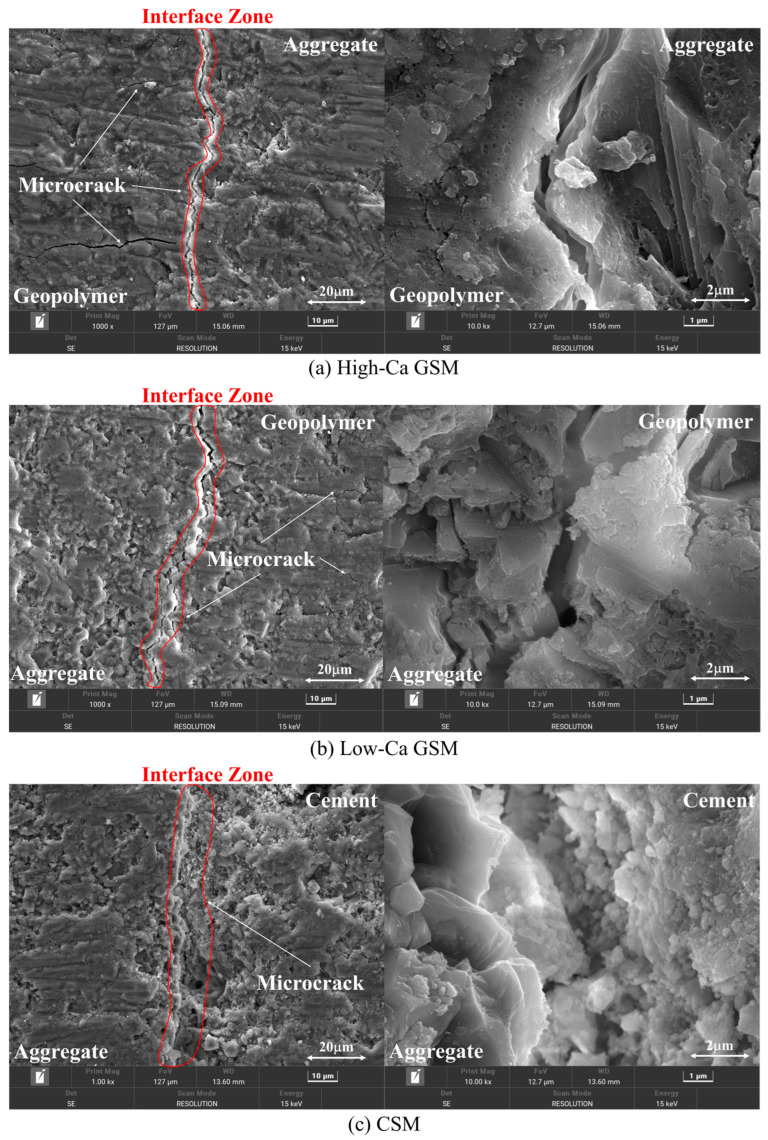
SEM image of the interface: (**a**) High-Ca GSM (G4); (**b**) Low-Ca GSM (M4); (**c**) CSM (P4).

To elucidate the bonding mechanism between geopolymer and aggregate and analyze the strength formation mechanism of GSM from a microscopic perspective, we conducted microscopic tests on the geopolymer–aggregate bonding interface, with a control group set up for the cement–aggregate bonding interface. Figure 18 presents SEM images of the bonding interface at magnifications of 1 k and 10 k.

**Figure 19:**
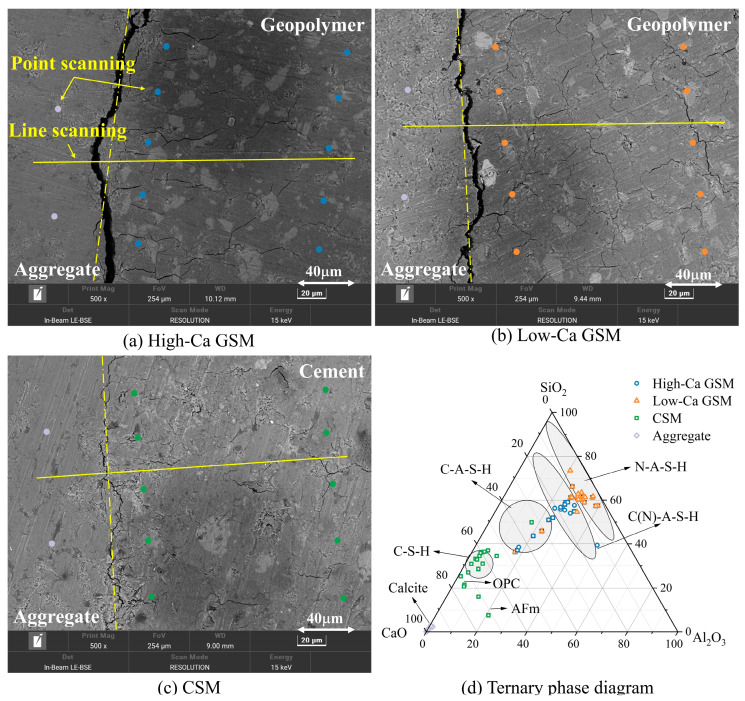
BSE image of the interface: (**a**) High-Ca GSM (G4); (**b**) Low-Ca GSM (M4); (**c**) CSM (P4); (**d**) Ternary phase diagram of Al_2_O_3_–SiO_2_–CaO in the interface.

At the interface, it is evident that the geopolymer in Figure 18a,b exhibits a denser structure compared with the cement in Figure 18c. The surface of the geopolymer appears blocky and smoother, with a reticulated gel structure visible in the topographic image at a 10k magnification [53,54]. This mesh structure enhances the compactness of the geopolymer, facilitating the formation of a monolithic block structure. The continuous and dense nature of the geopolymer underpins its excellent properties. In contrast, the surface of the cement (Figure 18c) appears uneven with more defects. The cement structure is looser, with agglomerated particles visible in the 10k magnified morphology image, contributing to a less compact cement structure. At the geopolymer or cement–aggregate interface, microcracks are visible, indicating the presence of an interfacial transition zone (ITZ) between the geopolymer or cement and aggregate. This region serves as a weak point in the strength of GSM or CSM. Figure 19 shows the BSE image of the interface region, revealing that high-Ca geopolymer contains more gel-like substances and unreacted particles. This is due to the rapid formation of C–A–S–H gel, which not only fills the structure but also adsorbs onto the surface of the reactive particles, limiting further reaction progress [51,55].

To investigate the chemical products at the interface, EDS point scanning was performed in the interface region, with points indicated in Figure 19. The EDS results revealed that the geopolymer is rich in Ca, Si, Al, and Na, while the cement is rich in Ca, Si, and Al, and the aggregate is primarily rich in Ca. Based on the point scanning results, a normalized Al_2_O_3_–SiO_2_–CaO ternary phase diagram of the interface products was plotted, as shown in Figure 19. In low-Ca geopolymer systems, the main interface product is N–A–S–H gel, while in high-Ca geopolymer systems, it is C–A–S–H gel, and in cement, the primary interface product is C–S–H gel [56,57]. The aggregate is mainly composed of CaCO_3_. N–A–S–H gel is also present in high-Ca geopolymer systems, and its mutual filling with C–A–S–H gel enhances the structural density, explaining why high-Ca geopolymer systems exhibit excellent fatigue performance [58].

**Figure 20:**
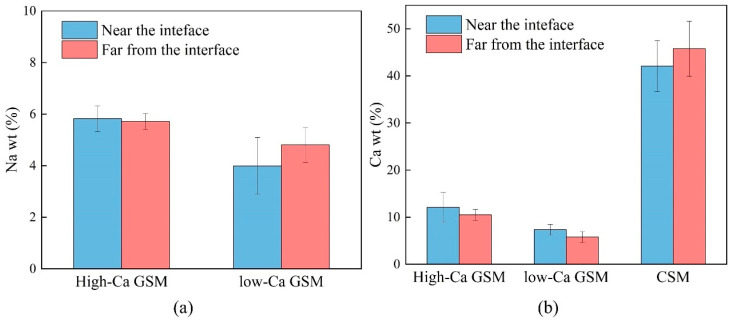
Variation in elemental content at the interface: (**a**) Na content of high-Ca GSM (G4) and low-Ca GSM (M4); (**b**) Ca content of high-Ca GSM (G4), low-Ca GSM (M4), and CSM (P4).

A graph depicting the variation in Ca and Na content at the interface products was plotted to further observe the chemical products at the interface, as shown in Figure 20. The Ca content increases near the geopolymer–aggregate interface but decreases near the cement–aggregate interface, suggesting that more C–A–S–H gel accumulates near the geopolymer interface, contributing to increased interface strength. Additionally, the Na content decreases in low-Ca geopolymer at the interface, while it increases in high-Ca geopolymer. This may be due to the higher amount of C–A–S–H gel in high-Ca geopolymer systems, which requires more N–A–S–H gel for mutual filling and forming C(N)–A–S–H gel [58].

**Figure 21:**
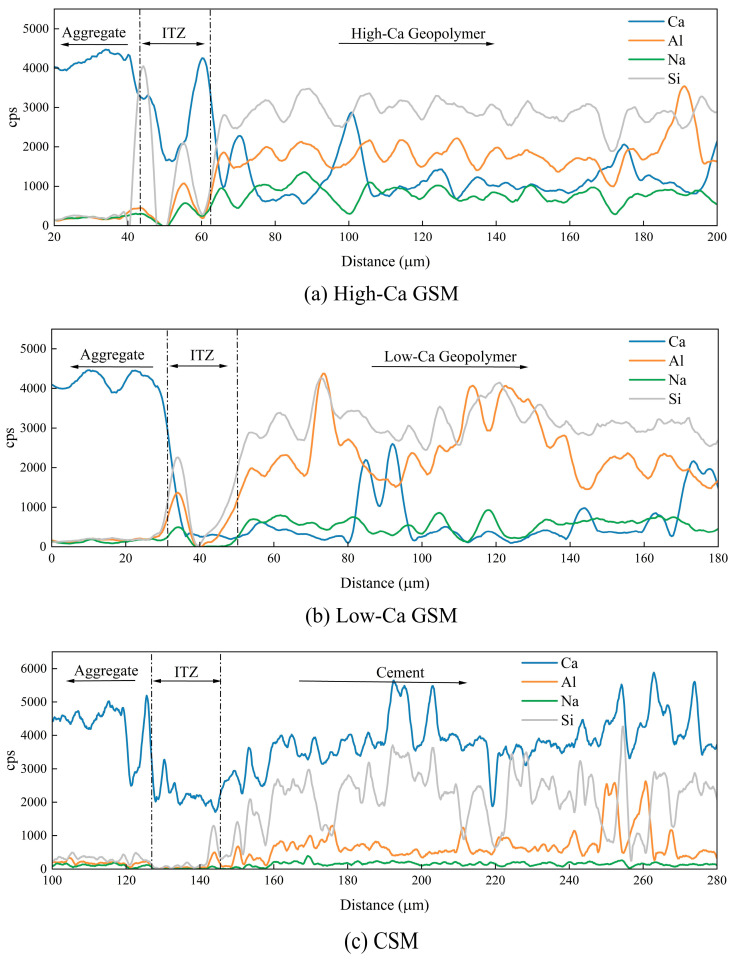
Distribution of elements in the interface: (**a**) High-Ca GSM (G4); (**b**) Low-Ca GSM (M4); (**c**) CSM (P4).

**Figure 22:**
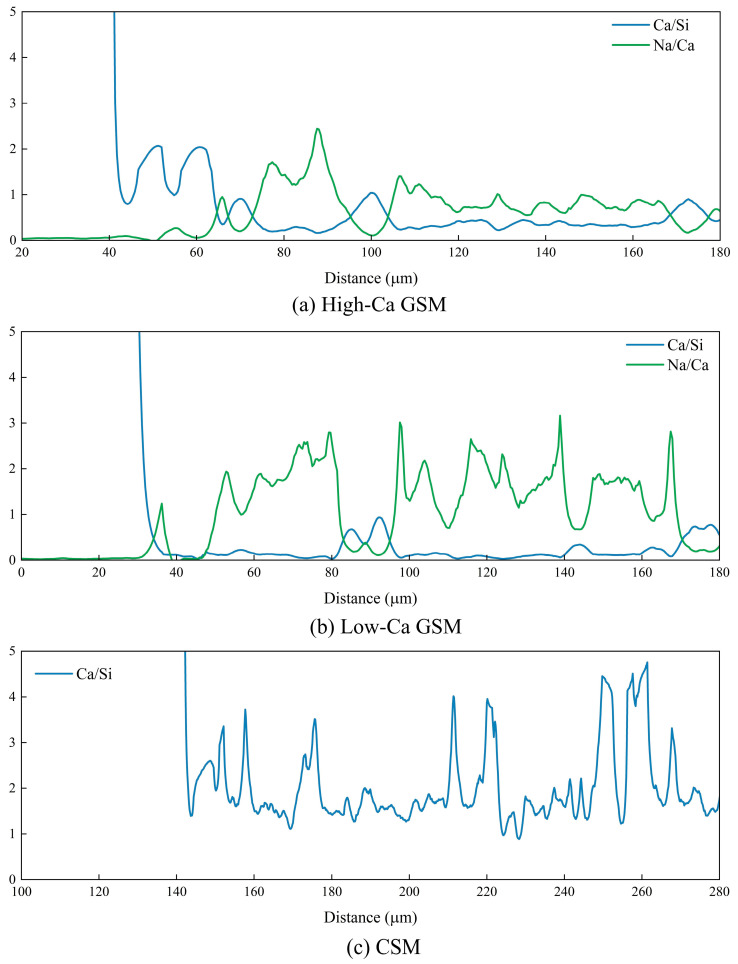
Ca/Si and Na/Ca in the interface: (**a**) High-Ca GSM (G4); (**b**) Low-Ca GSM (M4); (**c**) CSM (P4).

To study the distribution of elements perpendicular to the interface, EDS line scanning was performed in the interface region, with scanning positions indicated in Figure 19. Figure 21 shows the results of element concentration distribution obtained from the line scanning. Preliminary analysis revealed that the left side corresponds to the aggregate and the right side to the geopolymer or cement. A significant decrease in element concentration at the interface was observed, suggesting the presence of microcracks, with the primary source of interface bonding strength provided by mechanical interlocking. The width of these interface microcracks is approximately 20 μm, and the performance differences between geopolymer and cement are not caused by these microcracks. The Ca/Si and Na/Ca change curves at the interface, shown in Figure 22, indicate that the Ca/Si ratio of geopolymer or cement remains stable. However, an increasing trend in Na/Ca ratio is observed in high-Ca geopolymer systems near the interface region (62 μm–100 μm), suggesting an accumulation of C(N)–A–S–H gel near the interface. Dan et al. stabilized RAP using slag-based geopolymer, and through molecular dynamics simulation and microscopic tests, they also observed the accumulation of C(N)–A–S–H gel at the interface [59]. The interface products of high-Ca GSM, including C–A–S–H gel and C(N)–A–S–H gel, provide stronger mechanical interlocking, accounting for their excellent mechanical strength and fatigue performance.

## 4. Conclusions

This study investigated the macroscopic and microscopic mechanical properties of MK-GGBS-based geopolymer-stabilized macadam (GSM), including UCS, ITS, and fatigue life tests. The tests considered the effects of OPC, GGBS, and MK proportions, as well as the binder-to-aggregate ratio, to examine the mechanical properties of high-Ca GSM, low-Ca GSM, and cement-stabilized macadam (CSM). FTIR, SEM, and EDS tests were also conducted to analyze the micro-mechanisms underlying strength formation in these stabilized materials. The main conclusions are as follows:

- The mechanical properties of high-Ca GSM significantly improve with an increase in the binder-to-aggregate ratio, as high-Ca GSM forms C–A–S–H gel, which promotes rapid strength development.
- High-Ca GSM exhibits the highest fatigue life and resistance to permanent deformation under cyclic loading, surpassing both CSM and low-Ca GSM. Increasing the binder-to-aggregate ratio further enhances the density of high-Ca GSM, thereby improving its fatigue life and resistance to cyclic loading-induced permanent deformation.
- FTIR tests find that high-Ca GSM dissolves more [AlO_4_]^5−^ during the reaction compared with low-Ca GSM and CSM, resulting in the formation of more uniform gel and enhancing early strength development.
- SEM and EDS tests find that an aggregation of C(N)–A–S–H gel at the geopolymer-aggregate interface in high-Ca GSM, and the primary source of interface bonding strength is provided by mechanical interlocking in GSM and CSM.

## Figures and Tables

**Table 1:** Chemical compositions of the raw materials (Wt.%).

Oxides	SiO_2_	Al_2_O_3_	Fe_2_O_3_	CaO	MgO	K_2_O	SO_3_	Na_2_O	LOI *
MK	61.8	32.5	0.8	0.2	2.1	0.6	0.1	0.1	1.8
GGBS	35.3	11.9	0.4	37.8	6.9	0.6	0.1	0.3	0.1

* The weight loss of ignition at 1000 °C.

**Table 2:** Physical properties of aggregates.

Property	Coarse Aggregate Size > 9.5 mm	Fine Aggregate Size < 9.5 mm	Test Methods [28]
Percentage content of needle and flake particles (%)	12.1	-	T 0312
Water absorption (%)	1.25	1.98	T 0307/T 0330
Specific gravity by volume (g/cm^3^)	1.50	1.49	T 0309/T 0331
Crushing value (%)	10.3	-	T 0316
Percentage content of needle and flake particles (%)	12.1	-	T 0312

**Table 3:** Mixture for geopolymer-stabilized macadam, by mass.

Mixture ID	P4	M4	G3	G4	G5
OPC (%)	100	0	0	0	0
GGBS (%)	0	40	60	60	60
MK (%)	0	60	40	40	40
Binder to aggregate ratio	0.04	0.04	0.03	0.04	0.05
Optimum moisture content (%)	5.23	5.73	4.83	4.91	4.98
Maximum dry density (g/cm^3^)	2.366	2.356	2.346	2.388	2.422

## Data Availability

The data are contained within the article and/or are available on request from the corresponding author.
